# Evolutionary conserved role of neural cell adhesion molecule-1 in memory

**DOI:** 10.1038/s41398-020-00899-y

**Published:** 2020-07-06

**Authors:** Vanja Vukojevic, Pavlina Mastrandreas, Andreas Arnold, Fabian Peter, Iris-T. Kolassa, Sarah Wilker, Thomas Elbert, Dominique J.-F. de Quervain, Andreas Papassotiropoulos, Attila Stetak

**Affiliations:** 1 University of Basel, Department of Psychology, Division of Molecular Neuroscience, Birmannsgasse 8, CH-4055 Basel, Switzerland; 2 University of Basel, Department Biozentrum, Life Sciences Training Facility, Klingelbergstrasse 50-70, CH-4056 Basel, Switzerland; 3 University of Basel, Psychiatric University Clinics, Wilhelm Klein-Strasse 27, CH-4012 Basel, Switzerland; 4 University of Basel, Transfaculty Research Platform, Birmannsgasse 8, CH-4055 Basel, Switzerland; 5 Ulm University, Clinical & Biological Psychology, Institute for Psychology & Education, Albert-Einstein-Allee 47, D-89069 Ulm, Germany; 6 University Bielefeld, Department for Psychology and Sports Science, P.O. Box 100131, D-33501 Bielefeld, Germany; 7 University of Konstanz, Clinical Psychology & Behavioural Neuroscience, D-78457 Konstanz, Germany; 8 University of Basel, Department of Psychology, Division of Cognitive Neuroscience, Birmannsgasse 8, CH-4055 Basel, Switzerland

**Keywords:** Comparative genomics, Epigenetics and behaviour, Long-term memory

## Abstract

The neural cell adhesion molecule 1 (NCAM-1) has been implicated in several brain-related biological processes, including neuronal migration, axonal branching, fasciculation, and synaptogenesis, with a pivotal role in synaptic plasticity. Here, we investigated the evolutionary conserved role of NCAM-1 in learning and memory. First, we investigated sustained changes in ncam-1 expression following aversive olfactory conditioning in *C. elegans* using molecular genetic methods. Furthermore, we examined the link between epigenetic signatures of the NCAM1 gene and memory in two human samples of healthy individuals (*N* = 568 and *N* = 319) and in two samples of traumatized individuals (*N* = 350 and *N* = 463). We found that olfactory conditioning in *C. elegans* induced ncam-1 expression and that loss of ncam-1 function selectively impaired associative long-term memory, without causing acquisition, sensory, or short-term memory deficits. Reintroduction of the *C. elegans* or human NCAM1 fully rescued memory impairment, suggesting a conserved role of NCAM1 for memory. In parallel, DNA methylation of the NCAM1 promoter in two independent healthy Swiss cohorts was associated with memory performance. In two independent Sub-Saharan populations of conflict zone survivors who had faced severe trauma, DNA methylation at an alternative promoter of the NCAM1 gene was associated with traumatic memories. Our results support a role of NCAM1 in associative memory in nematodes and humans, and might, ultimately, be helpful in elucidating diagnostic markers or suggest novel therapy targets for memory-related disorders, like PTSD.

## Introduction

The fundamental process of memory formation involves several steps ranging from structural and functional remodeling of synapses to changes in gene expression and de novo protein synthesis^1–3^. Over the last decades, it became clear that both remodeling and formation of new synapses, where neuronal cell adhesion molecules play a critical role, are essential for memory. Neuronal cell adhesion molecules of the immunoglobulin superfamily, including L1 (L1 cell adhesion molecule) and NCAM1 (neuronal cell adhesion molecule 1), are known to shape the neuronal network during development and to be involved in cognitive functions and memory in different model organisms^4–7^. NCAM1 protein is predominantly localized at synaptic junctions where it contributes to the modulation of neuronal activity by altering the morphology and strength of synaptic connections^8^. This is particularly crucial during brain development where expression of cell adhesion molecules maintains the balance between stabilization and elimination of synapses.

The NCAM1 extracellular part consists of five Ig-like and two fibronectin type III homology domains and mediates both homophilic and heterophilic interactions. In the vertebrate nervous system, three different forms of NCAM1 are produced by alternative splicing of a single gene (NCAM-120, NCAM-140, and NCAM-180)^9,10^. The three NCAM1 isoforms differ in their intracellular part and exhibit distinct expression pattern and functions^11^. NCAM1 has been shown to be involved in both short- and long-term synaptic plasticity^4,12,13^. The role of NCAM1 in memory was first proposed by a pioneering study, which demonstrated that administration of antibodies or synthetic peptides against NCAM1 inhibited the induction of LTP^14^. Later studies demonstrated, that NCAM1 knockout (KO) mice show severe spatial memory deficits^15,16^ and loss of NCAM1 impaired fear conditioning^16,17^. Moreover, increasing amount of evidence points to the pivotal role of NCAM1 in the acquisition and formation of emotional memories (reviewed in refs. ^1–3,6^). Neuronal activity in the amygdala, a brain region centrally involved in emotional processing, is upregulated in response to stress in NCAM1 KO mice versus wild-type controls^4–7,18^. Moreover, NCAM1 seems to be required for fear conditioning and consolidation, as auditory and contextual fear memories were significantly impaired in NCAM1 KO mice^8,16^. In rats, expression of hippocampal NCAM1 changed upon emotional experience in a time- and intensity-dependent manner. Finally, exposure of rats to a traumatic event significantly impaired spatial memory formation and induced a reduction of the NCAM1 180 kDa isoform in the hippocampus^9,10,19^.

The broad range of biological effects of NCAM1 requires precise regulation of its protein activity. While post-translational modifications of NCAM1 have been widely studied, much less is known about the transcriptional regulation of NCAM1 in biological processes, including learning and memory. A recent study investigating gene expression activation during long-term associative memory (LTAM) in *C. elegans* identified the *NCAM1* homolog as one of the genes significantly upregulated in LTAM^20^. In addition, the promoter region of the human *NCAM1* was previously shown to contain a high proportion of CpG sites and to lack active TATA or CCAAT as transcriptional regulatory elements^21^, suggesting that methylation could be an important mechanism for the regulation of this gene’s expression. Generally, DNA methylation seems to be centrally involved in memory coding^22–27^, formation as well as maintenance^15,16,23–27^.

In the current study, we investigated the role of neural cell adhesion molecule 1 in learning and memory in nematodes and humans. We found that *ncam-1* is upregulated at the transcriptional level during a LTAM task in *C. elegans*. *ncam-1* loss of function *(lf)* specifically impaired LTAM, which was fully rescued by introduction of the human NCAM1 in mutant worms, suggesting an evolutionary conserved function of NCAM1 in long-term memory. Finally, we showed an association of *NCAM1* DNA methylation patterns with memory performance and traumatic memory, in healthy young individuals and conflict zone survivors, respectively.

## Methods and materials

### General methods and strains used

Standard methods were used for maintaining and manipulating *C. elegans*^28^ (see Supplemental Information).

### Targeted modification of *ncam-1* using CRISPR/Cas9

Loss of function mutant *ncam-1(utr3)* was generated using the CRISPR/Cas9 strategy, targeting two cleavage sites flanking the second intron of the gene (common to all three *C. elegans ncam-1* isoforms, see Supplemental Information).

### Extrachromosomal transgenic lines

For the *C. elegans* rescue experiment, *ncam-1(utr3)* mutant worms were injected with the 17.44 kb Eco47III/KpnI digested fragment from the pCC1FOS_ wrm0619dG03 fosmid. For the human rescue construct, human *ncam1* cDNA (encoding amino acids 1–858) was introduced under the control of a 2 kb *C. elegans ncam-1* promoter (see Supplemental Information).

### *C. elegans* behavioral assays

Chemotaxis to olfactory cues was tested as previously described^29^. Negative olfactory conditioning was performed with diacetyl (DA) as previously described^30^. LTAM was tested using two cycles of conditioning with 30 min feeding without DA inbetween trainings. After the spaced training, worms were kept on NGM plates in the presence of abundant food for 24 h and tested for their chemotaxis toward DA after the recovery phase^31^. For details see Supplemental Information.

### Real-time quantitative polymerase chain reaction (PCR)

Total RNA was isolated from synchronized adult worms using the Direct-zol RNA MiniPrep kit (Zymo Research Cooperation, Irvine, CA). Real-time PCR was performed with gene specific primers using the SyBr Fast kit (Kapa Biosystems,Wilmington, MA) according to the manufacturer’s recommendations in a Rotor Gene-6000 instrument (Corbett Research, Mortlake, NSW). Expression levels were normalized to *tba-1* expression level. Fold differences were calculated using the ΔΔCt method^32^ (see Supplemental Information).

### Fluorescence microscopy

Whole worms were mounted on 3% agar pads and immobilized with CTX buffer supplemented with sodium azide. NCAM-1::YFP animals were imaged using a Zeiss Axiovert 200 M LSM 5 Pascal confocal microscope equipped with a ×40 oil immersion objective (see Supplemental Information).

### Human studies

#### Swiss samples, healthy young adults

Memory was assessed in two independent samples of healthy young adults from Basel, Switzerland (Swiss Sample 1: *N* = 568; mean age 23.8 y, 18.3–36.8 y; 59% females; Swiss sample 2: *N* = 319; mean age 24.1 y, 18.3–36.5 y; 70% females). Subjects performed several different consecutive tasks as described in detail previously^33,34^. For the purpose of the present study we focused on episodic memory and therefore we analyzed the emotional and neutral picture-encoding task. In the Swiss sample 1, the delayed recognition task was tested 1h after encoding. In Swiss sample 2, the delayed free recall task was tested 24 h after encoding. For DNA isolation, blood samples were collected at the time-point of the main investigation. For details see Supplemental Information.

#### African samples, conflict survivors

PTSD risk and symptomatology were assessed in two independent African samples. In the African sample 1, we have investigated *N* = 350 survivors from the 1994 Rwandan genocide (mean age 34.8 y, 18–68 y; 49.1% females; 67.8% with PTSD lifetime diagnosis; Supplemental Table S1). For the African sample 2, we have included *N* = 463 survivors of the rebel war in Northern Uganda (mean age 29 y, 18–55 y; 44.1% females; 68.2% with PTSD lifetime diagnosis; Supplemental Table S1).

All subjects had experienced traumatic situations and were examined according to DSM-IV criteria^35^ in the period 2006–2009 (African sample 1) and 2009 to 2011 (African sample 2). Traumatic load was estimated by assessing the number of different traumatic event types experienced or witnessed^36^. Taking into account known ceiling effects of trauma load on PTSD risk, individuals with extreme levels of trauma exposure were excluded for the current analyses^37^. Saliva samples for the DNA isolation were collected at the time-point of the main investigation (see Supplemental Information).

### DNA isolation from human samples

Saliva samples were collected using an Oragene DNA Kit (DNA Genotek, Ottawa, ONT) and DNA was extracted using the precipitation protocol recommended by the manufacturer and then re-purified.

Blood samples were collected using the 10.0 mL BD Vacutainer^®^ Plus plastic whole blood tube, BD Hemogard^™^ closure with spray-coated K2EDTA (Becton Dickinson, Franklin Lakes, NJ). DNA was isolated with QIAmp Blood Maxi Kit (Qiagen AG, Hilden, Germany), using the recommended spin protocol (see Supplemental Information).

### Illumina human Methylation BeadChip methylation analyses

DNA isolated from peripheral blood or saliva was investigated with the Illumina human Methylation BeadChip 450 K array (Swiss Samples 1 & 2 and African Sample 1) or EPIC array (African Sample 2, restricted to the probe-set common with the 450 K array) (Illumina, Inc., San Diego, CA).

For preprocessing, data were extracted and analyzed using the R package RnBeads version 0.99.9^38^. The background was subtracted using the “noob” method in the methylumi package^39^, and the signal was further normalized using the SWAN algorithm^40^. Post-processing was further done for each of the four samples separately, combining the *B* values of the pre-processed data of all batches per sample (see Supplemental Information).

Finally, we used the genome-wide functional segmentation as specified by the ENCODE Combined chromatin states^41,42^, and then calculated mean methylation values for each of 13 functional elements of the *NCAM1* locus (GRCh37/hg19; rtracklayer R package^43^).

### Genotyping

Single-nucleotide polymorphisms (SNP) genotyping for all samples was done with *Affymetrix SNP 6.0* array platform for all four investigated samples, as previously described^44^. Quantitative trait loci (QTL) analysis was performed with MOLGENIS meQTL (methylation QTL) pipeline. For details see Supplemental Information.

### Statistical analyses

Delayed recognition or delayed recall as dependent variable was modeled against the DNA methylation of predefined functional ENCODE elements by linear regression, taking into account the interaction with the valence of pictures used in the emotional picture-encoding task.

In the African sample, the association between lifetime post-traumatic stress disorder symptom scores as dependent variables and DNA methylation of predefined functional ENCODE elements was assessed by linear regression. To account for trauma load as a principal factor in the development of PTSD^37^ sum of lifetime traumatic event types was used as a covariate in the linear regression model. The relationship between DNA methylation at *NCAM1* putative promoter and lifetime PTSD was assessed using binary logistic regression, with *NCAM1* promoter methylation as a quantitative predictor and sum of life traumatic event types as a covariate.

Bonferroni correction was implemented to account for multiple testing procedures. The significance threshold was set to *P* = 0.05. Statistical analyses were done in R (R version 3.6.0; R Development Core Team 2017), using the *cpg.assoc*^45^ and *nlme*^46^ R packages.

All human laboratory procedures were conducted in a blind, randomized order. For details see Supplemental Information.

## Results

### *ncam-1* is transcriptionally upregulated during LTAM

**Fig. 1:**
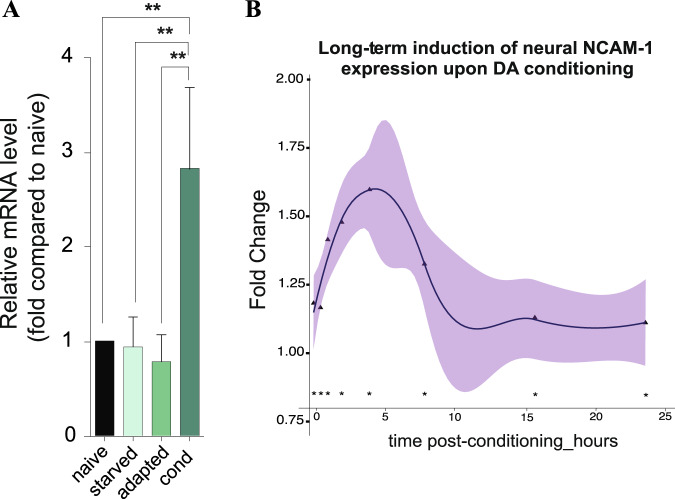
NCAM-1 is upregulated during long-term memory formation. **a** Relative *ncam-1* mRNA levels were measured using qRT-PCR from total RNA isolated from wild-type animals prior to treatment (naive), starved, DA only exposed and 4 h post conditioning (five biological samples each performed in triplicates). **b** Temporal transcriptomic analysis of *ncam-1* expression up to 24 h upon aversive conditioning, with peak expression occurring at 4 h post-conditioning (three independent biological replicates^20^).

In previous array-based experiments in *C. elegans*, *ncam-1* was upregulated during LTAM formation in a temporal manner^20,47^. To confirm this observation, we carried out qRT-PCR and assessed gene expression in trained worms (4 h after conditioning) compared to untrained worms or animals exposed to starvation or to DA alone. We found that *ncam-1* mRNA levels were significantly increased in trained animals (Fig. 1a). Furthermore, by using the temporal transcriptomic data from our recent publication^20^ we showed that *ncam-1* has a transcriptional peak 4 h after aversive olfactory conditioning training and remains upregulated up to 24 h post-conditioning (Fig. 1b).

### Loss of *ncam-1* impairs negative olfactory long-term memory

In order to study the physiological role of the sole *C. elegans* NCAM1 ortholog, we first generated a deletion in the *ncam-1* gene using the CRISPR/Cas9 system (Supplemental Fig. S1). Worms were injected with a mixture containing two different sgRNAs targeting distinct cleavage sites in the *ncam-1* gene locus and screened for deletion in the *ncam-1* gene. After screening we identified an allele carrying a deletion and simultaneous insertion that causes a frame shift in all three isoforms of the *C. elegans ncam-1* (Supplemental Fig. S1). Deletion of the *ncam-1* gene in worms does not result in a visible phenotype, animals appear healthy, are fertile and have no obvious morphological or locomotory defects.

**Fig. 2:**
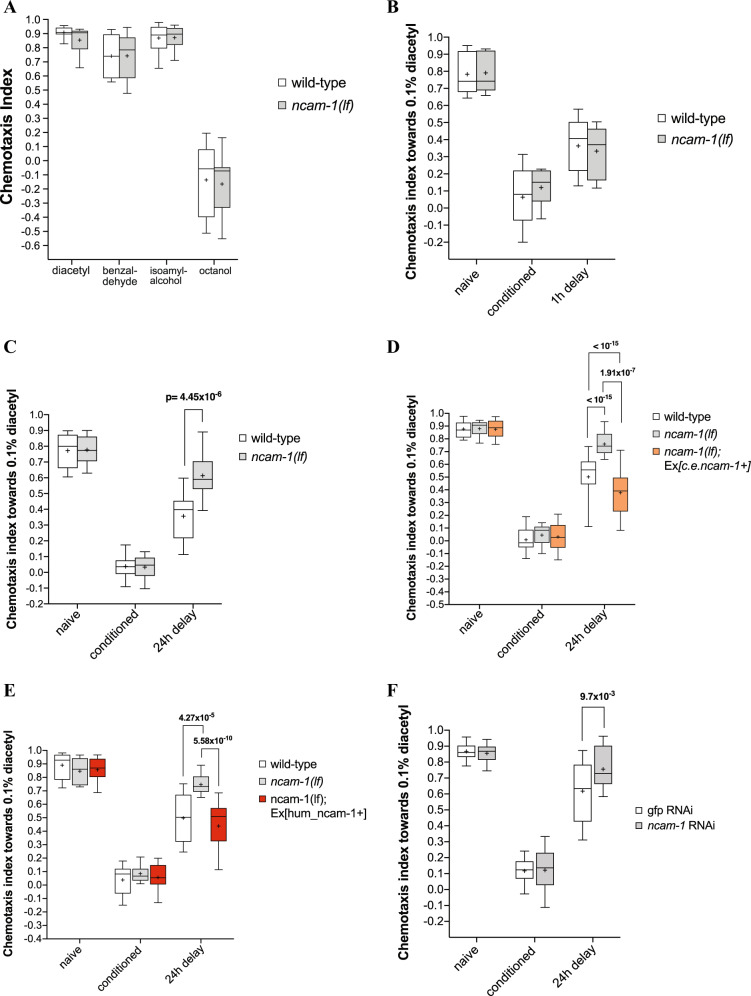
NCAM-1 regulates aversive long-term memory. **a** Chemotaxis of wild-type or mutant worms was assayed towards 0.1% diacetyl, benzaldehyde, or isoamyl-alcohol volatile chemoattractant, and 0.1% octanol as repellent. Chemotaxis index was calculated as CI= (worms at the attractant spot-worms at the solvent spot)/total number of worms. **b**, **c** Conditioned wild-type and *ncam-1(lf)* mutant worms were tested for their preference towards diacetyl immediately after conditioning (conditioned), after a 1 h (1 h delay), or after a 24 h delay (24 h delay). **d**, **e** LTAM DA conditioning of wild-type *ncam-1(lf)* worms carrying *C. elegans* genomic ncam-1 locus (**d**) or human NCAM1 cDNA (**e**) to DA, immediately (conditioned) or following 24-h recovery in the absence of DA (24 h delay). **f** LTAM conditioning of RNAi-hypersensitive worms treated with *ncam-1* or control *gfp* RNAi from early L3 until adulthood. Worms were assayed toward DA without (naive), immediately (conditioned) or 24 h following DA conditioning (24 h delay). All experiments were done in triplicate and repeated at least three times. Box plots showing the 10th and 90th percentiles are presented for each condition and genotype. Statistical significance was assessed with two-way ANOVA and post hoc *t* tests between groups as indicated (Bonferroni's adjusted *p* values are reported).

In order to test the role of *ncam-1* in the regulation of aversive olfactory associative learning, we first tested the chemotactic behavior of *ncam-1(lf)* mutant worms towards three different chemoattractants (diacetyl, isomyl-alcohol, and benzaldehyde) and a repellent (octanol) as previously described^29^ (Fig. 2a). Mutant worms exhibited similar chemotaxis to wild-type animals, indicating that *ncam-1(lf)* has no sensory defects. Next, we investigated the potential role of NCAM-1 in olfactory associative learning. In negative olfactory conditioning, untrained wild-type, and *ncam-1(lf)* animals exhibited strong attraction to DA to the same extent. Furthermore, after aversive olfactory conditioning with starvation in the presence of DA, both strains displayed strong repulsion toward DA, suggesting that *ncam-1* is not required for memory acquisition in this model. We also tested the ability of animals to retain this learnt behavior over time (short-term associative memory [STAM] or long-term associative memory [LTAM]). In STAM, nematodes were subjected to conditioning and allowed to recover for 1 h, prior to testing for their DA preference^48,49^. In both wild-type and *ncam-1(lf)* animals, the negative association of DA with starvation persisted to a similar extent during the recovery period tested (Fig. 2b). This result indicates that NCAM-1 is not required for aversive olfactory learning and for short-term memory in *C. elegans*. To test for LTAM, worms were conditioned using two rounds of training with food withdrawal in the presence of DA as described and were then tested for their DA attraction after a 24 h delay^24^. Whilst learning was similar between genotypes, a significant decrease in LTAM was observed in *ncam-1(lf)* mutants compared to wild-type worms (Fig. 2c). Reintroduction of a wild-type 18 kb genomic fragment of the *ncam-1* gene (Fig. 2d) or human *NCAM1* cDNA under the control of the *C. elegans* 5′ and 3′ regulatory elements into mutant worms fully rescued the memory defect (Fig. 2e). Finally, the effect of NCAM-1 was not due to a developmental defect, since RNAi silencing of *ncam-1* following neuronal differentiation phenocopied the *ncam-1(lf)* phenotype (Fig. 2f).

### Expression of NCAM-1 in *C. elegans*

**Fig. 3:**
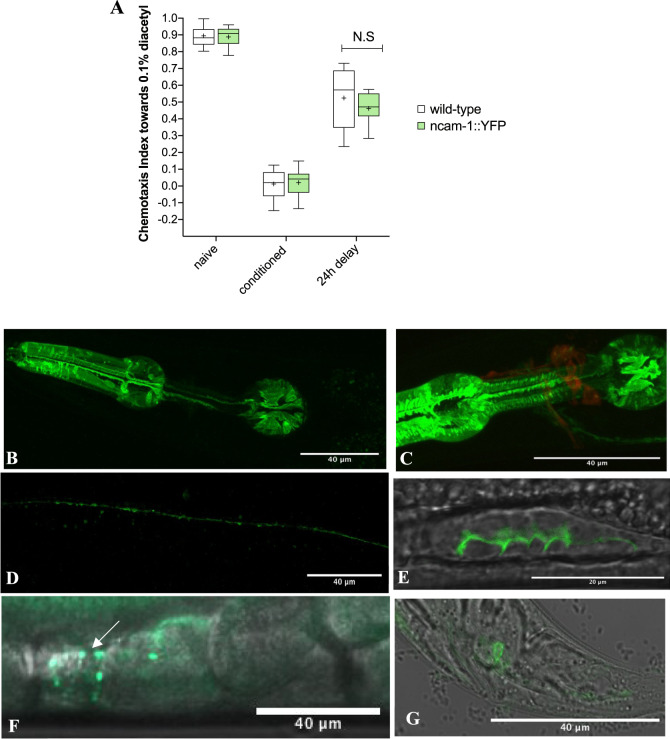
Expression pattern of NCAM-1. **a** Conditioned wild-type and *ncam-1::YPET* mutant worms were tested for their preference towards diacetyl immediately after conditioning (conditioned) and after a 24-h delay (24 h delay). All experiments were done in triplicate and repeated at least three times. Data sets were compared using two-way ANOVA. Box plots showing the 10th and 90th percentiles are presented for each condition and genotype. **b** Localization of *ncam-1::YPET* expression in the pharynx, **c** in the nerve ring (ncam-1 in green and DID stained head neurons in red), **d** in the VNC **e** in the germline, **f** in the spermatheca and **g** in the tail.

**Fig. 4:**
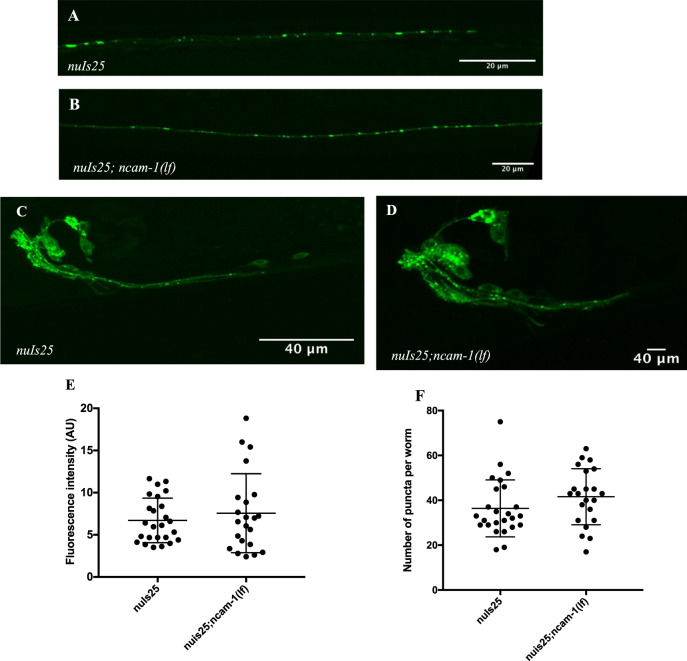
Synapse morphology in GLR-1 receptor subunits remains intact in *ncam-1 (lf)* worms. **a**, **b** Representative expression of GLR-1::GFP protein in the VNC in (**a**) *nuls25[glr-1::GFP]* and (**b**) *nuIs25[glr-1::GFP];ncam-1(lf)* transgenic worms. Images were always captured from the vulva all the way to the tail neuron. **c**, **d** Representative expression of GLR-1::GFP protein in the head ganglia in (**c**) *nuls25* and (**d**) *nuls25, ncam-1(lf)* transgenic worms. **e**, **f** Scatter plots displaying fluorescent intensity values **e** and number of punctae **f** per worm with the line in the middle representing the mean and the whiskers representing ± SD. A two-tailed unpaired Mann–Whitney *U* test revealed no significant differences between *nuls25* and *nuIs25; ncam-1(lf)* lines.

To investigate the expression pattern of NCAM-1, we tagged the endogenous protein at the C-terminal end with yellow fluorescent protein (YPET) using CRISPR/Cas9 strategy. The targeted genomic position was shared between all three *ncam-1* isoforms, ensuring that all protein isoforms are tagged. To confirm the functional integrity of the tagged protein, we compared the memory performance between wild-type and NCAM-1::YPET animals and found no difference (Fig. 3a), suggesting that YPET does not alter the function of the protein. Next, we analyzed the expression of NCAM-1 and found that the protein is expressed throughout the life cycle of *C. elegans* in several tissues, including the pharynx (Fig. 3b), nerve ring (Fig. 3c), in the ventral nerve cord (VNC) (Fig. 3d), germline (Fig. 3e), spermatheca (Fig. 3f), and some tail neurons (Fig. 3g)^50^. Since NCAM1 plays a pivotal role in synaptic formation, maturation and maintenance, we investigated synapse morphology in *ncam-1(lf)* worms. Specifically, we looked for changes in GLR-1 containing synapses since glutamatergic signaling has been previously shown to be essential in modulating associative memory formation in *C. elegans*^51^. By visualizing the distribution of GFP-tagged GLR-1 receptors (GLR-1::GFP) in wild-type and *ncam-1(lf)* animals, we showed that in the VNC, GLR-1::GFP puncta shape and distribution appeared to be unchanged (Fig. 4a, b) and the cell bodies of head neurons showed similar GLR-1::GFP distribution (Fig. 4c, d). We further carried out quantification analyses by measuring the fluorescence intensity (Fig. 4e) and the number of puncta (Fig. 4f) along the VNC, from the vulva region all the way to the tail neuron and showed no differences between wild-type and *ncam-1(lf)* animals. Together, our results suggest that loss of NCAM-1 does not interfere with the distribution or number of GLR-1 receptors suggesting that our memory phenotype is indeed attributed to the loss of the protein rather than to a general synaptic defect.

### DNA methylation of the human *NCAM1* promoter is associated with delayed recognition performance

**Fig. 5:**
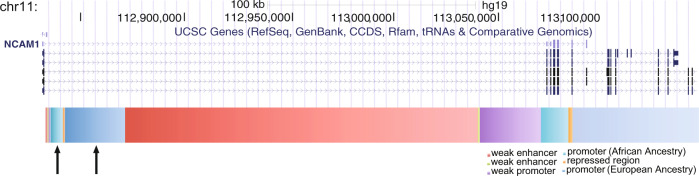
Genomic segmentation of the NCAM-1 locus. The genome-wide functional segmentation as specified by the ENCODE Combined chromatin states (GRCh37/hg19). The black arrows mark the two alternative *NCAM1* promoters associated to the populations of African and European descent, respectively.

Next, we investigated the association between DNA methylation of the functional elements of the human *NCAM1* gene locus and memory performance. We first used the Swiss sample 1, which was tested for delayed recognition of the previously seen emotional and neutral pictures. After accounting for multiple testing, we found that DNA methylation of the ENCODE-predicted *NCAM1* promoter (Fig. 5) was significantly associated with the delayed recognition performance (*β* = −0.46, *P*_uncorrected_ = 0.0004, *P*_corrected_ < 0.05, Supplemental Fig. S2A). Furthermore, there was a significant interaction between picture valence and DNA methylation of ENCODE-predicted *NCAM1* promoter (*F*_(2, 1114)_ = 5.7, *P*_interaction_ = 0.004). Post hoc analysis revealed that the DNA methylation of the *NCAM1* promoter was associated with the recognition performance of negative and neutral content, but not with the positive content (Table 1). Finally, DNA methylation of the *NCAM1* promoter was not associated with the immediate recall performance in the same emotional memory task (*P* > 0.05).

**Table 1:** Associations of putative *NCAM1* promoter methylation with delayed recognition and delayed recall memory in Swiss samples of healthy young individuals.

Sample	Valence	*t*-stat	*p* Value
*Swiss sample 1*	All pictures	−3.6	0.0004
Delayed recognition	Negative pictures	−1.9	0.041
Memory	Positive pictures	−0.9	0.359
(*N* = 568)	Neutral pictures	−2.9	0.004
*Swiss sample 2*	All pictures	−3.0	0.003
24 h delayed recall	Negative pictures	−2.7	0.006
Memory	Positive pictures	−1.8	0.077
(*N* = 319)	Neutral pictures	−0.6	0.572

### DNA methylation of the *NCAM1* promoter is associated with 24 h delayed free recall

In order to further investigate the association between *NCAM1* and remote memory performance we analyzed data of the Swiss Sample 2, in which we tested a 24-h delayed free recall of previously seen emotional and neutral pictures. Here, the DNA methylation of *NCAM1* promoter was negatively associated with the delayed recall of previously seen pictures (*β* = −3.0, *P*_uncorrected_ = 0.003). The interaction between valence and DNA methylation of the ENCODE-predicted *NCAM1* promoter on delayed free recall was nonsignificant (*F*_(2, 634)_ = 2.8, *P* = 0.06). However, a post hoc analysis revealed that the DNA methylation of the *NCAM1* promoter was associated with the delayed free recall of negative content, but not with the positive or neutral content (Table 1).

### DNA methylation of the *NCAM1* promoter is associated with lifetime intrusive memories

Long-lasting aversive memories of past traumatic events are an important cognitive component of posttraumatic stress disorder (PTSD) etiology and symptomatology. Guided by the findings in the healthy samples, we subsequently analyzed data of conflict zone survivors in the two African samples. First, a comparison of methylation levels across *NCAM1* Encode elements between the Swiss and African samples did not reveal significant differences in median, distribution, or variability (*P*_*s*_ > 0.05, see Methods). Next, given the known differences in the genetic architecture between populations of European and African descent, as well as the existence of alternative promoters of the *NCAM1* gene, we first performed an explorative analysis across all functional segments of the *NCAM1* locus (Fig. 5, Supplemental Fig. S2B). After accounting for multiple testing, we observed that the DNA methylation of the alternative, putative, *NCAM1* promoter was negatively associated with life-time PDS symptoms (main effect − African Sample 1: *t*_(347)_ = −3.0, *P* = 0.003, *P*_corrected_ < 0.05; and African sample 2: *t*_(460)_ = −3.3, *P* = 0.001), after taking trauma load into account. Specifically, after accounting for the experienced traumatic event types, the DNA methylation of this putative *NCAM1* promoter was negatively associated with lifetime intrusive memories of aversive events in both conflict zone survivor populations. On the other hand, the association with lifetime avoidance symptoms was significant only in African sample 1 and with lifetime hyperarousal symptoms only in African sample 2 (Table 2). In both African samples trauma load was positively associated with life-time PDS symptoms, as expected (*P*_*s*_ < 0.05), but had no significant effects on *NCAM1* promoter DNA methylation (*P*_*s*_ > 0.05).

**Table 2:** Associations of putative *NCAM1* promoter with PTSD symptoms and PTSD risk in African Samples of conflict zone survivors.

Sample	Phenotype	*t*-stat	*p* value
*African sample 1*	Avoidance	−2.4	0.019
(*N* = 350)	Intrusions	−2.1	0.001
	Hyperarousal	−0.5	0.601
	Sum	−3.0	0.003
	PTSD	−2.3	0.019
*African sample 2*	Avoidance	−1.7	0.089
(*N* = 463)	Intrusions	−2.7	0.029
	Hyperarousal	−3.5	0.0005
	Sum	−3.3	0.001
	PTSD	−0.9	0.118

**Fig. 6:**
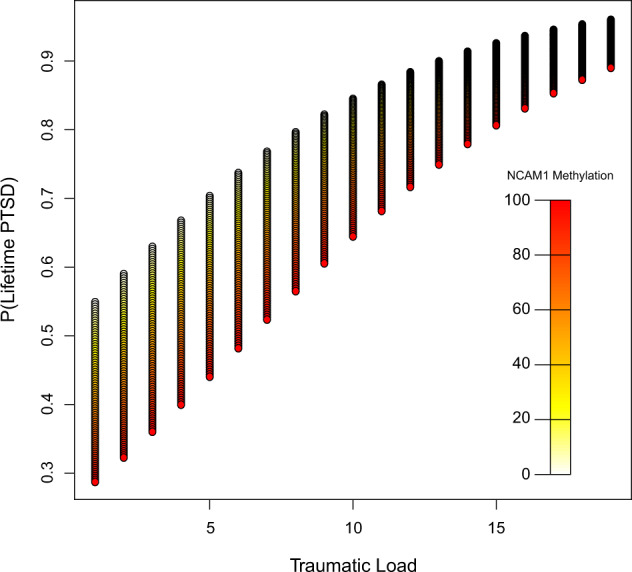
Fitted values for lifetime PTSD risk probability, dependent on trauma load and the *NCAM1* promoter methylation. Fitted values of the Lifetime PTSD risk derived from a binary logistic regression model with traumatic load and *NCAM1* promoter methylation as predictor variables. The range of the *NCAM1* promoter methylation (*NCAM1* methylation) is represented by heat colors, with white representing of 0% methylation (Beta = 0), and red representing 100% methylation (Beta = 1) (*N* = 347, African Sample 1).

Finally, we investigated whether methylation levels at the putative *NCAM1* gene promoter affected the association between trauma load and lifetime PTSD risk (see Materials and methods). We found that higher methylation levels were significantly associated with a lower lifetime PTSD risk in African Sample 1 (*t*_(347)_ = −2.3, Wald *x*^2^ = 4.3, *P* = 0.019; Fig. 6), while in the African sample 2 the association did not reach significance threshold (*t*_(460)_ = −0.9, *P* = 0.12; Table 2).

### Quantitative trait loci correlate with the variation of DNA methylation at the predicted *NCAM1* promoter regions

Analysis of the methylation QTLs (meQTL) associated with the *NCAM1* enhancer element methylation in the four cohorts revealed several in cis SNPs that significantly correlated with the variation of DNA methylation at the corresponding *NCAM1* promoter regions. The Supplemental Tables S2 and S3 summarize the shared meQTLs across all the samples of European and African descent, respectively.

## Discussion

The present study indicates that the neural cell adhesion molecule-1 is associated with associative long-term memory in nematodes and healthy humans, as well as with traumatic memories and PTSD risk in heavily traumatized conflict zone survivors.

Phenotypic analysis of the neural cell adhesion molecule-1 *(ncam-1)* deficient worms demonstrated a selective role of *ncam-1* for LTAM but not for learning (acquisition) or STAM. Previous studies in vertebrates have shown that NCAM1 is also essential in learning and short-term plasticity, particularly during the initiation phase of LTP^4,12,13^. However, the requirement of NCAM1 during learning has been mainly investigated in other model organisms using hippocampus-dependent tasks^52,53^, which would explain why in *C. elegans* NCAM-1 is not involved in memory acquisition.

Besides the role of NCAM1 in the learning process, antibody-driven inhibition of NCAM1 during the protein-synthesis-dependent phase of memory consolidation disrupts also the memory of the training experience^54,55^. Interestingly, a crucial period of 6–8 h post-passive-avoidance training is required for memory consolidation, which is in accord with the peak of transcriptional activation of *ncam-1*, 4 h after aversive olfactory conditioning training. Furthermore, *ncam-1* remained upregulated even 24 h after post-conditioning. These results suggest that transcriptional activation of NCAM-1 in the first 4–6 h could be important for long-term memory formation. Upregulation of NCAM-1 at the protein level following aversive olfactory conditioning would provide additional evidence, however, given the high endogenous expression of NCAM-1 in the pharyngeal tissue of the worms (Fig. 3b, c), we were unable to detect any changes coming from neural tissues (Supplemental Fig. S3). This was also paralleled by imaging results, where differences from the VNC, where most synapses are located, could not be detected due to high baseline signal (Supplemental Fig. S3).

Complex mechanisms are known to underlie memory both on the molecular as well as the anatomical level. In addition, precise temporal control of such processes is crucial for memory formation. Although our results (Fig. 1) suggest that transcriptional activation of NCAM-1 likely regulates its function during memory formation, we were not able to address the exact time window where such processes are essential.

The cellular function of NCAMs is to fine-tune the adhesive properties of cells and regulate the establishment of the cellular connectivity pattern in the brain^7,56,57^. During neurite outgrowth cells increase their migratory capacity by downregulating expression of cell adhesion molecules whilst once they innervate their target brain areas, their adhesiveness is upregulated, stabilizing the newly formed connections and locking cells into their final position^58^. Since NCAM1 expression is critical during nervous system development, memory deficits due to lack of NCAM1 could be related to the elimination of the protein during a critical developmental period and unrelated to its absence in adulthood. Indeed, by tagging the endogenous protein in *C. elegans*, NCAM-1 was expressed throughout the lifecycle of the worm, pointing to a continuous requirement for the gene, both in neuronal and nonneuronal tissues. These findings are supported by a previous report investigating the expression pattern of NCAM-1 with the help of a transcriptional reporter system^50^. However, given the strong functional redundancy between members of the IgCAM family^50^, loss of NCAM-1 function during development might be compensated, leading to proper contact and communication among neurons during synaptogenesis. In line with that, our RNAi experiment revealed that the memory phenotype was not due to a developmental defect, as L3 worms whose nervous system is established with a *ncam-1* knockdown phenocopied ncam-1 *(lf)*. In addition, ionotropic glutamate-receptor (GLR-1) localization studies revealed no detectable deficits in synapse morphology or distribution.

Furthermore, given the sequence homology shared between *C. elegans* and human NCAM-1, the conserved functional role observed across species, as well as the fact that the introduction of the human homolog rescued the memory defect of *ncam-1(lf)* in worms, prompted us to investigate the role of NCAM-1 in human memory.

In the present study we show that epigenetic signatures of the *NCAM1* gene promoter were associated with episodic memory performance in two independent samples of European descent. Moreover, in two populations of conflict zone survivors of African descent, DNA methylation at an alternative, putative promoter of the *NCAM1* gene was negatively correlated with lifetime intrusive memories of aversive events, after taking trauma load into account. Interestingly, interindividual differences in DNA methylation levels were not related to traumatic load in traumatized individuals. Furthermore, we did not observe significant differences in median, distribution, or variability of *NCAM1* promoter methylation between conflict zone survivors and healthy individuals nonexposed to trauma. These findings suggest that *NCAM1* methylation differences might pre-exist the conflict-related traumatic events. The pre-existing differences in DNA methylation may be a result of interplay between selected types of early adversity and specific timing of exposure^59,60^, may reflect other types of risks associated with the HPA axis inhibition^61,62^, or alternatively might be a result of development/early life associated stochastic epigenetic variation^63^. The profound role of NCAM1 for neuronal development during the perinatal period of life might offer a plasticity window that could significantly impact how we process and store experiences later in life. However, underpinning the casual pathways of early set epigenetic modifications, as well as their relative stability and reversibility requires further research.

Recently, we have shown that epigenetic regulation of glucocorticoid receptor gene is associated with memory processing in healthy individuals, but also plays an important role for the risk and pathogenesis of PTSD risk^24^. Glucocorticoid signaling is a key mediator of the effects of stressful learning on long-term memory facilitation by modulation of the consolidation processes. In synergy with processes initiated by learning, stress and glucocorticoid signaling transiently activates the expression of NCAM in memory-associated brain regions, thus facilitating the formation of long-term memories^64^. As mentioned, NCAM1 is involved in acquisition and formation of emotional memories (reviewed in ref. ^6^) and accordingly, is dysregulated in several neuropsychiatric disorders, such as schizophrenia, mood and anxiety disorders, or Alzheimer’s disease^65^.

In the present study, DNA was isolated from whole blood in the Swiss samples and from saliva in the African samples. Although the blood cells are of mesodermal origin, and the source of DNA in saliva is a mixture of ectodermal epithelial cells and mesodermal white blood cells^66^, we did not observe significant differences in the *NCAM1* methylation levels across the four samples investigated in this study. In addition, in a subset of European individuals with available DNA both from saliva and blood, *NCAM1* methylation did not significantly differ across the tissues^44^. Previous studies suggested that DNA methylation in CG-rich genomic regions (e.g., *NCAM1* promoter) generally show more stable epigenetic signatures across brain and non-brain tissues^67–71^. Finally, in the current study, several in cis meQTLs were associated with epigenetic signatures of the predicted *NCAM1* promoters, thus suggesting that these epigenetic marks share a degree of similarity across the tissues. These findings suggest that it might be possible, to a certain extent, to use non-brain tissue for the investigation of brain-related traits, such as psychiatric disorders.

Taken together, our results support a conserved role of NCAM1 in memory from nematodes to humans. This knowledge might prove helpful in elucidating novel therapies in the treatment of memory-related disorders.

## Supplemental Information

Supplemental Information
