# Supplementary material for: Evolutionary conserved role of neural cell adhesion molecule-1 in memory
Source: Transl Psychiatry. 2020 Jul 6;10:217. doi: 10.1038/s41398-020-00899-y (PMC7338365; doi:10.1038/s41398-020-00899-y)
Supplement: Supplementary file 1 — Supplemental Information [file 41398_2020_899_MOESM1_ESM.docx]

**Supplemental Information**

**Supplemental Methods and Materials**

**General methods and strains used**

Standard methods were used for maintaining and manipulating *C. elegans* (1). Common reagents were obtained from Sigma (Sigma-Aldrich, St Louis, MO) unless otherwise stated. The *C. elegans* Bristol strain, variety N2, was used as the wild-type reference strain in all experiments. Alleles and transgenes used were: *ncam-1(utr3), ncam-1(utr3); utrEx75[ncam-1 genomic locus, sur-5p::dsRed], ncam-1(utr3); utrEx76[C.e.ncam-1p::human ncam1 cDNA, sur-5p::dsRed], ncam-1(utr14[ncam-1::YPET::3xFlag]), sid-1(pk3321) V; uIs69 V. [pCFJ90 (myo-2p::mCherry) + unc-119p::sid-1], nuIs25 [glr-1p::glr-1::GFP + lin-15(+)], nuIs25;ncam-1(utr3).*

**Targeted modification of ncam-1 using CRISPR/Cas9**

Loss of function mutant *ncam-1(utr3)* was generated using the CRISPR/Cas9 strategy, targeting two cleavage sites flanking the second intron of the gene (common to all three *C. elegans ncam-1* isoforms). The obtained mutant carries a deletion and insertion in the targeted region causing premature stop and frameshift (Figure S1)

Endogenous tagging of *ncam-1* with YPET was generated as described previously (2). 446bp and 404bp homology arms flanking the C-terminus of *ncam-1* were PCR amplified from pCC1FOS_wrm0619dG03 fosmid and inserted into the mNG^SEC&3xFlag vector pDD283 using NEBuilder Hifi DNA assembly (New England Biolabs, Ipswich, MA). The Cas9 target site was selected using the MIT CRISPR design tool (<http://crispr.mit.edu>) and inserted into pDD162 (3). The sgRNA sequence used was 5’-TGCTCGATTCAAGTGGAGCTGTTTTAGAGCTAGAAATAGCAAGT-3’. A mixture of 50 ng/µl Cas9–sgRNA plasmid, 10 ng/µl repair template, and 2.5 ng/µl pCFJ90, 5 ng/µl pCFJ104 and 10 ng/µl *sur-5p::dsRed* co-injection markers was injected into the gonads of young adults (4). Knock-in line was established, SEC cassette was excised using heat shock and the *ncam-1* YPET line was sequenced to verify correct insertion of the tag.

**Extrachromosomal transgenic lines**

For the *C. elegans* rescue experiment, *ncam-1(lf)* mutant worms were injected with the 17.44 kb Eco47III/KpnI digested fragment from the pCC1FOS_ wrm0619dG03 fosmid. For the human rescue construct, human *ncam1* cDNA (encoding amino acids 1-858) designed with XhoI and EcoRV compatible ends, was placed under the control of a 2 kb *C. elegans* *ncam-1* promoter and fused with a 1 kb *C. elegans* *ncam-1* 3’UTR. T4 DNA ligase (New England Biolabs, Ipswich, MA) was used to assemble all the fragments and the final construct was verified by sequencing and injected into *ncam-1(lf)* worms. All transgenic lines were generated by injecting DNA at a concentration of 100 ng/μl into both arms of the syncytial gonad of worms as described previously (4). *sur-5p::mDsRed* was used as a transformation marker at a concentration of 10 ng/μl.

***C. elegans* behavioral assays**

All experiments were conducted with synchronized populations of one day old young adult hermaphrodites at 20°C. Chemotaxis to olfactory cues was tested as described (5). Briefly, a population of well-fed, young adults was washed three times with CTX buffer (5 mM KH_2_PO_4_/K_2_HPO_4_ pH 6.0, 1 mM CaCl_2_, and 1 mM MgSO_4_) and 50–200 worms were placed in the middle of a 10-cm test plate. Worms were given a choice between a spot of attractant or repellent diluted in ethanol with 1 µl 20 mM sodium-azide and a counter spot with ethanol and sodium-azide. The distribution of the worms over the plate was determined after 1 h and the chemotaxis index was calculated as previously described (Chemotaxis Index= $\frac{compound spot-ethanol spot}{total amount of worms on plate}$ ) (5). Negative olfactory conditioning was performed as previously described (6). Briefly, well-fed young adult worms were starved in the presence of 2 µl- undiluted chemoattractant (DA) for 1 h on 10 cm CTX plates (5 mM KH_2_PO_4_/K_2_HPO_4_ pH=6.0, 1 mM CaCl_2_, 1 mM MgSO_4_, 2% agar). Following conditioning, worms were tested for their chemotaxis towards DA either immediately following training to assess learning, or 1 h later to assess short-term associative memory. Long-term associative memory was tested using two cycles of conditioning with 30 min feeding without DA between trainings. After the spaced training, worms were kept on NGM plates in the presence of abundant food for 24 h and tested for their chemotaxis towards DA after the recovery phase (7).

**Real-time qPCR**

Total RNA was isolated from synchronized adult worms using the Direct-zol RNA MiniPrep kit (Zymo Research Cooperation, Irvine, CA) with DNase treatment and the mix was reverse-transcribed using a mix of random decamers (Ambion, Foster City, CA) and anchored oligo(dT)_20_ primers (Invitrogen, Carlsbad, CA). Real-time PCR was performed with gene specific primers (forward primer: GTTGGAATTATTCACCCACCAACTC, reverse primer: GAATTGATACTCCGTTGGCCTC) using the SyBr Fast kit (Kapa Biosystems, Wilmington, MA) according to the manufacturer’s recommendations in a Rotor Gene-6000 instrument (Corbett Research, Mortlake, NSW). Expression levels were normalized to *tba-1* (forward primer: CTGCTGACAAGGCTTACCATG, reverse primer: CAAACAGCCATGTACTTTC) expression level. Fold differences were calculated using the ΔΔCt method (8).

**Fluorescence Microscopy**

Whole worms were mounted on 3% agar pads and immobilized with CTX buffer supplemented with sodium azide. NCAM-1::YFP animals were imaged using a Zeiss Axiovert 200 M LSM 5 Pascal confocal microscope equipped with a 40x oil immersion objective at different larval stages and as young adults. For identification of amphid and phasmid neurons, worms were co-stained with DiD (ThermoFisher Scientific, Waltham, MA) at a final concentration of 10 µg/µl. YPET was excited using the 488-nm argon laser and detected using LP525-550 emission filter. DiD was excited using the 633-nm helium-neon laser and detected using LP650 emission filter. For imaging of the *nuls25* and *nuls25; ncam-1(lf)* lines, only synchronized one-day old adult worms were used and animals were grown at temperatures of 15, 20 and 25 °C. Animals were imaged using a Zeiss 880 laser scanning confocal microscope equipped with a 63x oil immersion objective. GFP was excited using the 488-nm argon laser and detected using LP525-550 emission filter. Images were processed and quantified using ImageJ. For the quantification of fluorescence intensity, the VNC region between the vulva and the tail neuron were imaged using identical settings. On average, three to four images were acquired per worm. The mean gray value was calculated for each image and normalized to its corresponding background. All mean gray values were then summed up and normalized to the total length of each animal so as to correct for length differences between worms. For the puncta number calculation, the multipoint tool was used.

**Protein extraction and western blot analysis**

For protein extraction, worms were collected in ice-cold RIPA buffer (50mM Tris-HCl pH 7.5, 150mM NaCl, 1% Triton-X-100, 0.5% sodium-deoxycholate, 0.1% SDS, 1mM EDTA, 10 mM NaF, 1 mM Na-orthovanadate) supplemented with protease inhibitor cocktail (Roche, Basel, Switzerland). Samples were homogenized in Mixer Mill MM 301 (Retsch GmbH, Germany) for 30s repeated four times. Lysates were cleaned by centrifugation at 13.000 rpm for 20 min at 4°C. Protein concentration of the supernatant was measured using Pierce BCA Protein Assay Kit (ThermoFisher Scientific, Waltham, MA) according to the manufacturer’s instructions.Samples were subjected to SDS-PAGE, transferred to PVDF membranes, blocked with 5% non-fat dry milk in TBST (50mM Tris-HCl, pH 7.5, 150mM NaCl, 0.05% Tween-20) and incubated with primary antibodies as indicated. Antibodies used were: mouse anti-FLAG (1:1000, Sigma Aldrich, St. Louis, MI) and mouse anti-actin (1:2000, Merck Millipore, Burlington, MA). Primary antibodies were detected using HRP coupled secondary antibodies (1:5000, Jackson ImmunoResearch Laboratories, Cambridge House, UK). Chemiluminescent signal was developed using Clarity and ClarityMax Western Blotting Substrates (BioRad Laboratories Inc., Hercules, CA) followed by detection with a FujiFilm ImageQuant LAS-4000 detector (GE Healthcare, Chicago, IL).

**Human studies**

Swiss Samples, healthy young adults

Memory was assessed in two independently recruited samples of subjects who participated in ongoing behavioural and imaging genetics studies of healthy, young adults in the city of Basel, Switzerland (Swiss Sample 1: N=568; mean age 23.8 y, 18.3-36.8 y; 59% females; Swiss Sample 2: N=319; mean age 24.1 y, 18.3-36.5 y; 70% females; data lock April 2015). The investigation was carried out in accordance with the latest version of the Declaration of Helsinki. The ethics committee of the Cantons of Basel-Stadt and Basel-Landschaft approved the experiments. All participants received general information about the study and gave their written, informed consent for participation. Participants were healthy, free of any neurological or psychiatric illness, and did not take any medication at the time of the experiment (except hormonal contraceptives).

Subjects performed several different consecutive tasks as described in detail previously

(9, 10). For the purpose of the present study we focused on episodic memory and therefore we analyzed the emotional and neutral picture-encoding task. Briefly, stimuli in the picture-encoding task consisted of 72 pictures selected from the International Affective Picture System (11) as well as from in-house standardized picture sets that allowed us to equate the pictures for visual complexity and content (e.g., human presence). On the basis of normative valence scores (from 1 to 9), pictures were assigned to emotionally negative (2.3 ± 0.6), emotionally neutral (5.0 ± 0.3), and emotionally positive (7.6 ± 0.4) conditions, resulting in 24 pictures for each emotional valence. After encoding, participants gave a free recall of the pictures in a separate room (no time limit was set for this task, same in both Swiss samples). Approximately 60 min after the presentation of the last picture in the encoding task, participants performed a recognition task for 20 min. (Swiss Sample 1; delayed recognition). The recognition task consisted of two sets of stimuli that were either new (i.e., not presented before) or old (i.e., presented during the picture- encoding task). Each of the two sets contained 72 pictures (24 pictures for each emotional valence). In Swiss Sample 2 a delayed free recall task was tested 24 hours after encoding (delayed recall). No recognition task was performed in this study sample. For DNA isolation, saliva samples were collected at the time-point of the main investigation.

African Samples, conflict survivors

PTSD risk and symptomatology were assessed in two independent African samples of conflict zone survivors. For the African sample 1 we included N = 350 survivors from the 1994 Rwandan genocide who lived as refugees in the Nakivale settlement in Uganda (mean age 34.8 y, 18-68 y; 49.1 % females; 67.8 % with PTSD lifetime diagnosis; 40.3 % subjects with current PTSD). For the African sample 2 we included 463 survivors of the rebel war in Northern Uganda. Data collection took place in the former internally displaced people (IDP) camps of Anaka, Pabbo (Amuru District) and Koch Goma (Nwoya District), and in resettled communities and villages of Gulu District, Northern Uganda (mean age 29 y, 18-55 y; 44.1 % females; 68.2 % with PTSD lifetime diagnosis; 17.4 % subjects with current PTSD).

All subjects had experienced traumatic situations and were examined according to DSM-IV criteria (12) in period 2006 to 2009 (African Sample 1) and 2009 to 2011 (African Sample 2). The Post-Traumatic Diagnostic Scale (PDS; (13)) was administered as a structured interview by expert psychologists from the University of Konstanz, Germany, as well as by trained local interviewers. The PDS was used to assess current and lifetime symptoms of intrusions, avoidance, and hyperarousal (Table S1) as well as the current and lifetime diagnosis of PTSD according to DSM-IV. Since spontaneous remission might have occurred during the time between the traumatic experiences and the interview (14), the lifetime variables were chosen as the main outcomes for the statistical analyses. In order to determine lifetime symptoms, participants were asked to report the symptoms according to the worst period of 4 weeks since the traumatic event. As it is difficult to respectively recall the severity of the symptoms, respondent where only asked if they experienced the respective symptom or not. Accordingly, the resulting lifetime scores represent the number of experienced symptoms, but not the symptom severity.

A checklist of 36 (African Sample 1, (15)) and 62 (African Sample 2,

(16)) war-related and general traumatic event types (e.g., injury by weapon, rape, accident) was used to assess traumatic events. Traumatic load was estimated by assessing the number of different traumatic event types experienced or witnessed, which is a reliable measurement of traumatic event exposure and showed the strongest relationship with lifetime PTSD

(16). To avoid known ceiling effects on PTSD risk - at extreme levels of trauma load, the probability of lifetime PTSD approaches 100%

(17), subjects were selected to have experienced up to 19 or 59 traumatic event types, for the African Sample 1 and African Sample 2, respectively. To exclude genetic relatives in the samples, only one person per household was interviewed. Additionally, given the genetic data was also available, IBD (identity by descent) threshold > 0.08 was applied. Candidates exhibiting current alcohol abuse and acute psychotic symptoms were excluded.

Saliva samples were collected at the time-point of the main investigation for the DNA isolation. Study procedures were approved by the ethics committees of the University of Konstanz, Germany; the Mbarara University of Science and Technology, Mbarara, Uganda; the Gulu University, Uganda; Lacor Hospital, Gulu, Uganda; and the Ugandan National Council for Science and Technology (UNCST), Uganda. Before the interview, all participants provided informed consent. For details see (18).

**DNA isolation from human samples**

Saliva DNA was collected in the African Sample using an Oragene DNA Kit (DNA Genotek, Ottawa, ONT) and initially extracted using the precipitation protocol recommended by the manufacturer. High-purity DNA was obtained by additional re-purification. For this purpose, 2µg of DNA isolated via the Oragene procedure was incubated overnight at 50°C with proteinase K (lysis buffer: 30 mM Tris-HCl pH 8.0, 10 mM EDTA, 1% SDS, 150 ng/l proteinase K), agitated by gentle orbital shaking. Next, the DNA was purified using a Genomic DNA Clean & Concentrator Kit (Zymo Research, Irvine, CA).

Blood samples were collected in the Swiss Samples from all subjects using the BD Vaccutainer Push Button blood collection set and 10.0 mL BD Vacutainer® Plus plastic whole blood tube, BD Hemogard™ closure with spray-coated K2EDTA (Becton Dickinson, Franklin Lakes, NJ). DNA was isolated from the remaining fraction, upon plasma removal. The isolation was performed with QIAmp Blood Maxi Kit (Qiagen AG, Hilden, Germany), using the recommended spin protocol. The DNA quality and concentration were assessed using spectrophotometry (Nanodrop 2000; ThermoScientific, Waltham, MA) and fluorometry (Qubit dsDNA BR Assay Kit, Invitrogen, Carlsbad, CA).

**Illumina human Methylation BeadChip methylation analyses**

DNA isolated from peripheral blood or saliva (for the African samples) was investigated with the 450 K array (Swiss Samples 1 & 2; African Sample 1) or EPIC array (African Sample 2, restricted to the probe-set common with the 450 K array) (Illumina, Inc., San Diego, CA). The subjects of the main Swiss sample (N = 568) were processed in two batches (2 plates and 4 plates). For the Swiss replication sample (4 plates, N = 319, African Sample 1 (4 plates, N = 350) and African Sample 2 (6 plates, N = 463) all subjects were processed in a single batch. Within a batch, samples were processed with a randomized plate assignment and with a single bisulfite conversion.

Preprocessing of data was done separately for each batch. Data were extracted and analyzed from the generated idat files using the R package RnBeads version 0.99.9 (19). CpG annotation was based on the manufacturer’s annotation file (Human-Methylation450_15017482_v.1.2). During preprocessing, the background was subtracted using the “noob” method in the methylumi package (20), and the signal was further normalized using the SWAN algorithm (21). The following probe categories were excluded from the final data sets, based on the annotation provided within the RnBeads package: non-CpG context probes (due to underrepresentation on the 450 K array, 0.6%, (22); functional differences when compared to the CpG context as well as very low abundance of non-CpG methylation in somatic tissues

(23) ; N = 3091); probes with a SNP mapping directly to the target CpG site, as well as probes with three and more SNPs mapping within the 50mer probe (see Supplementary Fig. 2; MAF threshold was set to 0.01; N = 18,998 CpGs); gonosomal probes (N = 11,473 CpGs); non-specific probes. Using the Greedycut algorithm, we iteratively removed the probes and data sets of the highest impurity (rows and columns in the detection p-value table that contain the largest fraction of unreliable measurements; p < 0.05; for each sample (19)).

Post-processing was further done for each sample separately, combining the B-values of the preprocessed data of all batches per sample. The B-values were further processed step-by-step in order to correct for further influential and putative confounding factors: 1) using logit- transformation (M-value,

(24), done with the R-package car (25)); 2) z-transformation per plate (correcting for plate and batch effects); 3) regressing out the first 8 (Swiss sample 1), 7 (Swiss sample 2) or 10 (African Samples) axes of a principal component analysis (PCA, done with the R-package pcaMethods

(26)). The PCA was based on CpGs with no missing values (>95% of the included CpGs). The PCA-based approach corrected for technical biases as well as for part of the variability induced by blood cell composition (European descent Samples); 4) regressing out the effects of sex and age; 5) regressing out the effects of variants in the 50mer probe sequence, if the total variance explained by these variants exceeded 0.1%.

The accepted missing rate per CpG was set to <1%. We further excluded cross-hybridizing probes and polymorphic CpG sites (27, 28) (Nmax = 63,974). Only CpGs surviving all filtering steps in all samples were used for the downstream analyses (N = 394,043).

Cell-count estimates of cell types (CD8+ T helper cells, CD4+ T helper cells, natural killer cells, B-cells, monocytes, granulocytes) were done with the minfi-package (29) in R, based on the algorithm provided by Houseman et al., 2012, adapted for the Illumina 450 K array (30). For details and validation of Infinium 450K array processing see (31).

Finally, we used the genome-wide functional segmentation as specified by the ENCODE Combined chromatin states (32, 33), and then calculated mean methylation values for each of the functional elements (GRCh37/hg19; rtracklayer R package (34)). The 13 functional elements of the *NCAM1* locus investigated in the current study are shown in the Figure 5.

***Affymetrix SNP 6.0 based genotyping***

SNP genotyping for all samples was done as previously described (32). Briefly, digestion was done with 250ng of DNA, in parallel with Sty I and Nsp I restriction enzymes (New England Biolabs). Following, enzyme specific adaptor oligonucleotides were ligated and material was amplified by PCR. 250μg of purified PCR was further used for the fragmentation with the average size of fragmentation products below 180bps. Labelled DNA was hybridized onto Genome-Wide Human SNP 6.0 Array (Affymetrix, Santa Clara, CA USA). Generation of SNP calls and array quality control were performed using the command line programs of the Affymetrix Power Tools package (version: apt-1-14.4.1). According to the manufacturer’s recommendation, Contrast QC was chosen as QC metric, using the default value of greater or equal than 0.4. All samples passing QC criteria were subsequently genotyped using the Birdseed (v2) algorithm. Mean Call Rate for was 98.7%. This value refers to per sample (i.e., individual) call rate and ranged from 90.1% to 99.7%. After basic SNP-QC (MAF > 0.02; HWE > 0.001; missing rate per SNP < 5%), *659,944* SNPs were used the further analyses (across 3 samples). Outliers were excluded by projecting the genotypic data on the two first PCA components inferred from HapMap reference populations (YRI, CEU and CHB-JPT populations), using a Bayesian Clustering Algorithm (35). *In cis* (< 25 kbp) quantitative trait loci (QTL) analysis was performed with MOLGENIS meQTL (methylation QTL) pipeline

(36).

**Statistical analyses**

Delayed recognition or delayed recall as dependent variable was assessed against the DNA methylation of predefined functional ENCODE elements by linear models, taking into account the interaction with the valence of pictures used in the emotional picture-encoding task. In the African sample, the association between lifetime post-traumatic stress disorder symptom scores as dependent variables and DNA methylation of predefined functional ENCODE elements was assessed by linear models. To account for trauma load as a principal factor in the development of PTSD

(17) sum of lifetime traumatic event types was used as a covariate in the linear regression model

(9). The relationship between DNA methylation at *NCAM1* putative promoter and lifetime PTSD was assessed using binary logistic regression, with *NCAM1* promoter methylation as a quantitative predictor and sum of life traumatic event types as a covariate.

A comparison of methylation levels of *NCAM1* Encode Elements between the Swiss and the African population was done by Kruskal–Wallis one-way ANOVA. Furthermore, we additionally assessed the equality of distributions and variability between the populations using Kolmogorov–Smirnov two-sample test and Siegel–Tukey test respectively. The the association between DNA methylation as dependent variable, and trauma load was assessed by linear models.

Bonferroni correction was implemented to account for multiple testing procedures. The significance threshold was set to P = 0.05. Statistical analyses were done in R (R version 3.6.0; R Development Core Team 2017), using the *cpg.assoc* (37) and *nlme* (38) R packages.

All laboratory procedures were conducted in a blind, randomized order, including DNA isolations, bisulfite conversion and DNA methylation analysis. Only after performing all procedures and excluding samples with low quality controls and outliers, further analysis with phenotypic data was performed.

For the *C. elegans* experiments, data was processed using Prism 7. Main effects and interaction terms were tested using ANOVA. Statistical tests for significance were done with F-tests using sum-of-squares type I. The p-value threshold was set to nominal significance (p < 0.05). In case of a significant main or interaction effect, significance between data was tested using post-hoc t-tests. P-values of the post-hoc tests were corrected for the number of tests calculated per analysis (Bonferroni-correction per analysis: p_bonf_ < 0.05). For the imaging quantification, the data was found to violate normality assumptions suggesting that the data followed a non-Gaussian distribution. Thus, non-parametric Mann-Whitney two-tailed unpaired U-tests were carried out to assess any differences between groups, with the p-value threshold set to nominal significance (p < 0.05)

**Supplemental Figures and Tables**

**Figure S1.** **Characterization of the *ncam-1(utr3)* deletion.**

**A**. The genomic sequence of *ncam-1* covering the deletion region. Position of the second intron common to all three isoforms is highlighted in blue and the PAM motifs recognised by the Cas9 are depicted in red. Orange highlights an additional insertion sequence in the *ncam-1(utr3).*

**B**. The amino-acid sequence of wild-type NCAM-1 compared to the mutant protein, with a premature STOP codon shown with an asterisk.

**Figure S2 DNA methylation of NCAM-1 Encode Functional Regions associations with delayed recognition performance / life-long PTSD symptoms.**

**A.** Association (–log_10_p) of DNA methylation across 13 ENCODE segments of the *NCAM1* locus and with delayed recognition performance of previously seen emotional pictures (*N =* 568, Swiss Sample 1). **B.** Association (–log_10_p) of DNA methylation across 13 ENCODE segments of the *NCAM1* locus with the sum of the lifetime PTSD symptoms according to the international PDS scale (*N =* 350, African Sample 1).

The ENCODE functional segments are aligned by genomic position (GRCh37/hg19). The horizontal full line indicates the *P <* 0.05 Bonferroni-corrected significance threshold.

**A**

**B**

**C**

0h


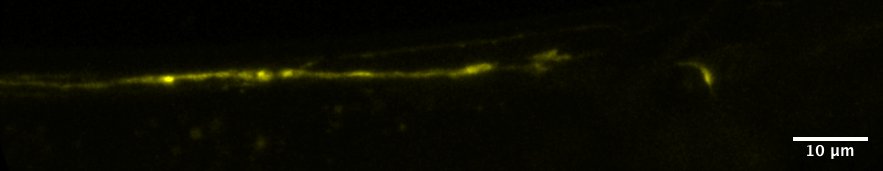

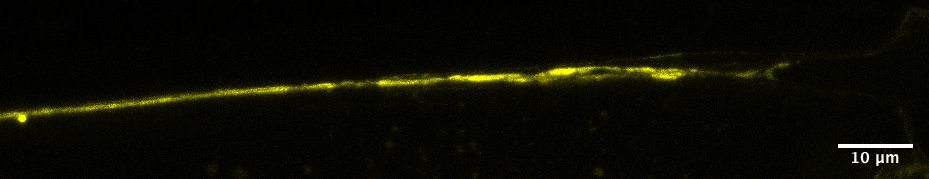


Naive


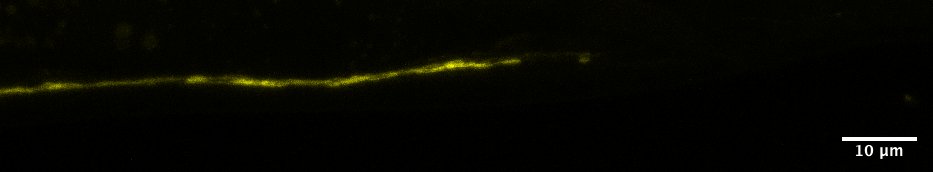


4h

**Ventral nerve cord**

**Tail**

**Figure S3 NCAM-1 protein changes following aversive olfactory conditioning.**

**A.** Representative western blot of the temporal expression of the NCAM-1::YPET::FLAG at naïve, 0h, 2h and 4h post-olfactory conditioning. For each condition, whole worm lysates were analysed using FLAG antibody (top). As the loading control, membranes were re-probed for actin (bottom). *Global naïve refers to the same NCAM-1::YPET lysate loaded across all western blots to normalize for differences in blocking, antibody affinity and exposure. **B.** Table displays quantification as a fold change to naïve levels, after normalization to global naïve and actin levels (data from 4 independent blots). **C.** Representative confocal microscopy images of the ventral nerve cord of NCAM-1::YPET animals tested at naïve level, immediately after conditioning (0h) and 4h after conditioning.

|  | Avoidance | Hyperarousal | Intrusions | PDS Sum | PTSD | Traumatic Event Types |
| --- | --- | --- | --- | --- | --- | --- |
| **African Sample 1** (*N = 350*) | 4.4 (0-7) | 3.5 (0-5) | 4.2 (0-5) | 11.7 (0-17) | 67.8% | 11.3 (0-19) |
| **African Sample 2** (*N = 463*) | 3.4 (0-7) | 3.1 (0-5) | 3.5 (0-5) | 10.0 (0-17) | 68.2% | 25.5 (3-59) |

**Table S1.** Ugandan and Rwandan Lifetime PDS Symptom Count Scores (mean, (min, max)) and Lifetime PTSD Diagnosis (%).

| Swiss Sample 1 | |  |  |  |  |  |  |  |
| --- | --- | --- | --- | --- | --- | --- | --- | --- |
| SNPName | SNPChr | SNPChrPos | CisTrans | SNPType | AlleleAssessed | Z_Score | Beta | P_Value |
| rs12575544 | 11 | 112918985 | cis | A/G | A | 15.806 | 0.598 | 2.84E-56 |
| rs10891495 | 11 | 112918941 | cis | C/T | C | 15.118 | 0.577 | 1.24E-51 |
| rs2155293 | 11 | 112901094 | cis | A/T | A | 9.286 | 0.377 | 1.60E-20 |
| rs17114705 | 11 | 112899832 | cis | A/G | A | 9.161 | 0.372 | 5.12E-20 |
| rs17114702 | 11 | 112899735 | cis | T/C | T | 9.102 | 0.371 | 8.84E-20 |
| rs17114689 | 11 | 112894450 | cis | A/G | G | 8.999 | 0.368 | 2.28E-19 |
|  |  |  |  |  |  |  |  |  |
| Swiss Sample 2 | |  |  |  |  |  |  |  |
| SNPName | SNPChr | SNPChrPos | CisTrans | SNPType | AlleleAssessed | Z_Score | Beta | P_Value |
| rs12575544 | 11 | 112918985 | cis | A/G | A | 12.359 | 0.621 | 4.38E-35 |
| rs10891495 | 11 | 112918941 | cis | C/T | C | 11.357 | 0.580 | 6.88E-30 |
| rs17114702 | 11 | 112899735 | cis | T/C | T | 8.444 | 0.451 | 3.08E-17 |
| rs2155293 | 11 | 112901094 | cis | A/T | A | 8.378 | 0.446 | 5.37E-17 |
| rs17114705 | 11 | 112899832 | cis | A/G | A | 8.378 | 0.446 | 5.37E-17 |
| rs17114689 | 11 | 112894450 | cis | A/G | G | 8.151 | 0.437 | 3.62E-16 |

**Table S2.** meQTLs (methylation Quantitative Trait Loci) associated with the DNA methylation at *NCAM1* predicted promoter in the European samples. meQTLs was performed across 2 European cohorts (N_Swiss Sample 1_ = 568, N_Swiss Sample 2_ = 319). Analysis revealed several *in cis* (<25 kbp) SNPs reaching genome-wide significance, with 6 meQTLs shared across the European cohorts.

| African Sample 1 | |  |  |  |  |  |  |  |
| --- | --- | --- | --- | --- | --- | --- | --- | --- |
| SNPName | SNPChr | SNPChrPos | CisTrans | SNPType | AlleleAssessed | Z_Score | Beta | P_Value |
| rs10750016 | 11 | 112837740 | cis | A/T | A | 9.767 | 0.477 | 1.56E-22 |
| rs720024 | 11 | 112838512 | cis | C/T | C | 6.536 | 0.330 | 6.33E-11 |
|  |  |  |  |  |  |  |  |  |
| African Sample 2 | |  |  |  |  |  |  |  |
| SNPName | SNPChr | SNPChrPos | CisTrans | SNPType | AlleleAssessed | Z_Score | Beta | P_Value |
| rs10750016 | 11 | 112837740 | cis | A/T | A | 5.142 | 0.372 | 2.72E-07 |
| rs720024 | 11 | 112838512 | cis | C/T | C | 4.498 | 0.330 | 6.85E-06 |

**Table S3.** meQTLs (methylation Quantitative Trait Loci) associated with the DNA methylation at *NCAM1* predicted alternative promoter in the African samples. meQTLs was performed across 2 African cohorts (N_African Sample1_ = 350, N_African Sample2_ = 463). Analysis revealed several *in cis* (<25 kbp) SNPs reaching genome-wide significance, with 2 meQTLs shared across the African cohorts.

**Supplemental References**

1. Brenner S (1974) The genetics of Caenorhabditis elegans. *Genetics* 77(1):71–94.

2. Dickinson DJ, Pani AM, Heppert JK, Higgins CD, Goldstein B (2015) Streamlined Genome Engineering with a Self-Excising Drug Selection Cassette. *Genetics* 200(4):1035–1049.

3. Dickinson DJ, Ward JD, Reiner DJ, Goldstein B (2013) Engineering the *Caenorhabditis elegans* genome using Cas9-triggered homologous recombination. *Nat Meth* 10(10):1028.

4. Mello CC, Kramer JM, Stinchcomb D, Ambros V (1991) Efficient gene transfer in C.elegans: extrachromosomal maintenance and integration of transforming sequences. *EMBO J* 10(12):3959–3970.

5. Bargmann CI, Hartwieg E, Horvitz HR (1993) Odorant-selective genes and neurons mediate olfaction in C. elegans. *Cell* 74(3):515–527.

6. Nuttley WM, Atkinson-Leadbeater KP, Van Der Kooy D (2002) Serotonin mediates food-odor associative learning in the nematode Caenorhabditiselegans. *Proc Natl Acad Sci USA* 99(19):12449–12454.

7. Vukojevic V, et al. (2012) A role for α-adducin (ADD-1) in nematode and human memory. *EMBO J* 31(6):1453–1466.

8. Pfaffl MW (2001) A new mathematical model for relative quantification in real-time RT-PCR. *Nucleic Acids Research* 29(9):e45.

9. de Quervain DJ-F, et al. (2007) A deletion variant of the alpha2b-adrenoceptor is related to emotional memory in Europeans and Africans. *Nat Neurosci* 10(9):1137–1139.

10. Heck A, et al. (2017) exome sequencing of healthy phenotypic extremes links TROVE2 to emotional memory and PTSD. *Nat hum behav* 1:0081.

11. Lang PJ, Bradley MM and Cuthbert BN (1997) International Affective Picture System (IAPS): Technical Manual and Affective Ratings. NIMH Center for the Study of Emotion and Attention, 39-58.

12. Association AP (2000) *Diagnostical and Statistical Manual of Mental Disorders, fourth edition (DSM-IV-TR)* (American Psychiatric Association, Washington, DC).

13. Foa EB, Cashman L, Jaycox L, Perry K (1997) The validation of a self-report measure of posttraumatic stress disorder: The Posttraumatic Diagnostic Scale. *Psychological Assessment* 9(4):445–451.

14. Kolassa I-T, et al. (2010) Spontaneous remission from PTSD depends on the number of traumatic event types experienced. *Psychological Trauma: Theory, Research, Practice, and Policy* 2(3):169–174.

15. Ertl V, et al. (2010) Validation of a mental health assessment in an African conflict population. *Psychological Assessment* 22(2):318–324.

16. Wilker S, et al. (2015) How to quantify exposure to traumatic stress? Reliability and predictive validity of measures for cumulative trauma exposure in a post-conflict population. *European Journal of Psychotraumatology* 6:28306.

17. Kolassa I-T, et al. (2010) Association study of trauma load and SLC6A4 promoter polymorphism in posttraumatic stress disorder: evidence from survivors of the Rwandan genocide. *The Journal of clinical psychiatry* 71(5):543–547.

18. Vukojevic V, et al. (2014) Epigenetic modification of the glucocorticoid receptor gene is linked to traumatic memory and post-traumatic stress disorder risk in genocide survivors. *Journal of Neuroscience* 34(31):10274–10284.

19. Assenov Y, et al. (2014) Comprehensive analysis of DNA methylation data with RnBeads. *Nat Meth* 11(11):1138–1140.

20. Davis S, Du P, Bilke S, Triche, JrT, Bootwalla M (2020). *methylumi: Handle Illumina methylation data*. R package version 2.34.0.

21. Maksimovic J, Gordon L, Oshlack A (2012) SWAN: Subset-quantile within array normalization for illumina infinium HumanMethylation450 BeadChips. *Genome Biol* 13(6):R44.

22. Bibikova M, et al. (2011) High density DNA methylation array with single CpG site resolution. *Genomics* 98(4):288–295.

23. Ziller MJ, et al. (2013) Charting a dynamic DNA methylation landscape of the human genome. *Nature* 500(7463):477–481.

24. Du P, et al. (2010) Comparison of Beta-value and M-value methods for quantifying methylation levels by microarray analysis. *BMC Bioinformatics* 11:587.

25. Fox J, Weisberg S (2011) *An R Companion to Applied Regression, Second Edition* (Thousand Oaks (CA)). SAGE.

26. Stacklies W, Redestig H, Scholz M, Walther D, Selbig J (2007) pcaMethods--a bioconductor package providing PCA methods for incomplete data. *Bioinformatics* 23(9):1164–1167.

27. Chen Y-A, et al. (2013) Discovery of cross-reactive probes and polymorphic CpGs in the Illumina Infinium HumanMethylation450 microarray. *Epigenetics* 8(2):203–209.

28. Price ME, et al. (2013) Additional annotation enhances potential for biologically-relevant analysis of the Illumina Infinium HumanMethylation450 BeadChip array. *Epigenetics & Chromatin* 13(1):R44.

29. Aryee MJ, et al. (2014) Minfi: a flexible and comprehensive Bioconductor package for the analysis of Infinium DNA methylation microarrays. *Bioinformatics* 30(10):1363–1369.

30. Jaffe AE, Irizarry RA (2014) Accounting for cellular heterogeneity is critical in epigenome-wide association studies. *Genome Biol* 15(2):R31.

31. Milnik A, et al. (2016) Common epigenetic variation in a European population of mentally healthy young adults. *Journal of Psychiatric Research* 83:260–268.

32. Dunham I, et al. (2012) An integrated encyclopedia of DNA elements in the human genome. *Nature* 489(7414):57–74.

33. Hoffman MM, et al. (2012) Unsupervised pattern discovery in human chromatin structure through genomic segmentation. *Nat Meth* 9(5):473–476.

34. Lawrence M, Gentleman R, Carey V (2009) rtracklayer: an R package for interfacing with genome browsers. *Bioinformatics* 25(14):1841–1842.

35. Bellenguez C, et al. (2011) A robust clustering algorithm for identifying problematic samples in genome-wide association studies. *Bioinformatics* 28(1):134–135.

36. van der Velde KJ, et al. (2019) MOLGENIS research: advanced bioinformatics data software for non-bioinformaticians. *Bioinformatics* 35(6):1076–1078.

37. Barfield RT, Kilaru V, Smith AK, Conneely KN (2012) CpGassoc: an R function for analysis of DNA methylation microarray data. *Bioinformatics* 28(9):1280–1281.

38. Pinheiro J, Bates D, DebRoy S, Sarkar D, R Core Team (2020). *nlme: Linear and Nonlinear Mixed Effects Models*. R package version 3.1-148
